# Supplementary material for: Azacitidine Plus Venetoclax for the Treatment of Relapsed and Newly Diagnosed Acute Myeloid Leukemia Patients
Source: Cancers (Basel). 2022 Apr 16;14(8):2025. doi: 10.3390/cancers14082025 (PMC9028084; doi:10.3390/cancers14082025)
Supplement: Supplementary file 1 [file cancers-14-02025-s001.zip › cancers-1651045-supplementary.pdf]

cancers

MDPI

Article

Azacitidine plus Venetoclax for the Treatment of Relapsed and Newly Diagnosed Acute Myeloid Leukemia Patients

Sylvain Garciaz [1,2,*], Marie-Anne Hospital [2], Anne-Sophie Alary [3], Colombe Saillard [2], Yosr Hicheri [2], Bilal Mohty [2], Jérôme Rey [2], Evelyne D'Incan [2], Aude Charbonnier [2], Ferdinand Villetard [2], Valerio Maisano [2], Laura Lombardi [2], Antoine Ittel [3], Marie-Joelle Mozziconacci [4], Véronique Gelsi-Boyer [4,5] and Norbert Vey [1,2,*]

Supplementary Table S1. Panel of genes Next-generation sequencing (NGS).

ABL1	***EZH2***	***PTPN11***
ANKRD26	*FBXW7*	*RAD21*
ASXL1	*FLT3*	*RhoA*
ASXL2	*GATA2*	*RIT1*
ATM	*HRAS*	*RUNX1*
BCOR	*IDH1*	*SETBP1*
BCORL1	*IDH2*	*SF3B1*
BRAF	*JAK2*	*SH2B3*
CALR	*KDM6A*	*SMC1A*
CBL	*KIT*	*SMC3*
CDKN2A	*KRAS*	*SRSF2*
CEBPalpha	*MPL*	*STAG2*
CREBBP	*NF1*	*STAT3*
CSF3R	*NOTCH1*	*SUZ12*
CSNK1A1	*NOTCH2*	*TET2*
CUX1	*NPM1*	*TNFAIP3*
DDX41	*NRAS*	TP53
DNMT3A	*PHF6*	*U2AF1*
ETNK1	*PPMD1*	*WT1*
ETV6	*PTEN*	*ZRSR2*

Supplementary Table S2. Response rates at Day-28 and Day-56.

	**Total**		**ND AML**		**R/R AML**	
**Response at day-28 (n=77)**	N	%	N	%	N	%
CR	**10**	13	**6**	16	**4**	10
CRi	**11**	14	**7**	18	**4**	10
PR	**2**	3	**1**	3	**1**	3
MLFS	**13**	17	**9**	24	**4**	10
SD/PD	**31**	40	**12**	32	**19**	49
death	**9**	12	**2**	5	**7**	18
unknown	**1**	1	**1**	3	-	-
Response at day-56 (n=77)	N	%	N	%	N	%
CR	**17**	22	**12**	32	**5**	13
CRi	**8**	10	**5**	13	**3**	8
PR	**1**	1	**0**	**-**	**1**	3
MLFS	**10**	13	**5**	13	5	13
SD/PD	**27**	35	**8**	21	**19**	49
death	**12**	16	**6**	16	**6**	15

unknown	**2**	3	**2**	5	**0**	0

Supplementary Table S3. Response rates at Day-28 and Day-56 in the group of the VIALE-A eligible patients.

		**1st line**	
**Response at day-28 (n=64)**		**N**	**%**
	CR	**4**	14
	CRi	**6**	21
	PR	**1**	3
	MLFS	**8**	28
	SD/PD	**8**	28
	death	**1**	3
	unknown	**1**	3
Response at day-56 (n=64)			
	CR	**8**	28
	CRi	**5**	17
	PR	**0**	0
	MLFS	**5**	17
	SD/PD	**5**	17
	death	**4**	14
	unknown	**2**	7

Supplementary Table S4. Response rates assessed at day-56 in the cytogenetics and molecular subgroups and according to VEN cycle 1 dose.

**cytogenetics**		**non-adverse**		**adverse**	
**Response at day-56 (n=77)**		**N**	**%**		**%**
	CR	**8**	20	**9**	25
	CRi	**5**	12	**3**	8
	PR	**1**	2	**0**	0
	MLFS	**9**	22	2	6
	SD/PD	**13**	32	**13**	36
	death	**4**	10	**8**	22
	unknown	**1**	2	**1**	3
Molecular subgroup		TP53/RAS WT		TP53/RAS mut	
Response at day-56 (n=54)					
	CR	**9**	28	**4**	18
	CRi	**5**	16	**2**	9
	PR	**0**	0	**0**	0
	MLFS	**7**	22	3	14
	SD/PD	**5**	16	**10**	45
	death	**4**	13	**3**	14
	unknown	**2**	6	**0**	0
VEN cycle 1 dose		>50%		≤50%	
Response at day-56 (n=54)		N	%		
	CR	**9**	18	**7**	32
	CRi	**6**	12	**2**	9
	PR	**0**	0	**1**	5
	MLFS	**8**	16	**3**	14
	SD/PD	**19**	37	**6**	27

death	**8**	16	**2**	9
unknown	**1**	2	**1**	5

Supplementary Table S5. Factors associated with response in the ND-AML group.

ND-AML	responders		dead/non responders		RR	P value
N, %	**22**	100	**14**	100		
male	**12**	55	**10**	71	0.76 (0.46-1.27)	0.48
age, median (range)	**74**	(61-81)	**72**	(67-79)		0.95
age >75 years	**7**	32	**6**	43	0.82 (0.46-1.48)	0.73
Secondary AML	**16**	73	**10**	71	0.92 (0.55-1.82)	1
AML-MRC	**10**	45	**3**	21		0.17
therapy-related	**2**	9	**2**	14		1
post MPN	**4**	18	**5**	36		0.26
Previous azacitidine	**2**	9	**3**	21	0.62 (0.21-1.87)	0.36
WBC, median (range)	**1.8**	(0.4-60)	**8.8**	(0.8-40)		0.47
WBC >10G/L	**7**	32	**5**	36	0.97 (0.55-1.73)	1
plt count, median (range)	**100**	(11-272)	**55**	(3-200)		0.18
plt <20G/L	**3**	14	**4**	29	0.65 (0.27-1.60)	0.39
BM blasts, median (range)	**36**	(7-70)	**36**	(12-88)		0.83
BM blasts <30%	**9**	41	**6**	43	0.97 (0.57-1.65)	1
Adverse cytogenetics	**13**	59	**8**	57	1.03 (0.61-1.76)	1
monosomal	**8**	36	**4**	29		1
complex	**7**	32	**6**	43		0.83
genomic alteration						
NPM1	**2**	14	**1**	7	1.13 (0.49-2.65)	1
FLT3	**1**	7	**1**	7	0.83 (0.2-3.4)	1
ITD	**0**	0	**0**	0		-
TKD	**1**	7	**1**	7		-
IDH (n=37)	**5**	23	**3**	21	1.02 (0.56-1.9)	1
IDH1	**4**	18	**3**	21		-
IDH2	**1**	5	**0**	0		-
TP53 (n=33)	**3**	14	**5**	42	0.52 (0.20-1.31)	0.10
JAK2 (n=31)	**2**	10	**5**	42	0.39 (0.12-1.31)	0.07
ASXL1 (n=28)	**7**	39	**5**	50	0.9 (0.49-1.63)	1
RUNX1 (n=28)	**4**	22	**4**	40	0.75 (0.35-1.6)	0.43
TET2 (n=28)	**3**	17	**4**	40	0.63 (0.25-1.55)	0.37
DNMT3A (n=28)	**2**	11	**3**	30	0.6 (0.2-1.8)	0.34
SFSR2 (n=28)	**4**	22	**3**	30	0.89 (0.44-1.84)	1
NRAS (n=28)	**4**	22	**3**	30		1
RAS/TP53 (n=28)	**4**	22	**7**	70	0.69 (0.33-1.46)	0.41
VEN dose >50% during cycle 1 (n=37)	**11**	52	**9**	69	0.86 (0.49-1.49)	0.72

Supplementary Table S6. Factors associated with response in the R/R-AML group.

R/R cohort	responding	death/non responding	RR	P value

N, %	**14**	100	**25**	100		-
male	**10**	71	**14**	56	1.56 (0.59-4.09)	0.49
age, median (range)	**75**	(22-86)	**65**	(22-77)		0.10
age >75 years	**7**	50	**2**	8	3.33 (1.59-6.97)	0.005
Secondary AML	**5**	36	**14**	56	0.58 (0.24-1.43)	0.32
AML-MRC	**4**	29	**8**	32		1
therapy-related	**0**	0	**3**	12		0.54
post MPN	**1**	7	**3**	12		1
Previous treatments						
median number of line	**2**	(1-3)	**1**	(1-4)		0.63
Azacitidine	**3**	21	**7**	28	0.71 (0.26-2.13)	0.72
median cycle (range)	**6**	(3-20)	**4**	(3-8)		0.22
chemotherapy	**14**	100	**20**	80	0.38 (0.39-15.5)	0.14
allogenic transplantation	**3**	21	**7**	28	0.79 (0.28-2.27)	0.72
WBC, median (range)	**2**	(0.4-13)	**4.5**	(7-73)		0.21
WBC >10G/L	**2**	14	**7**	28	0.56 (0.15-2.54)	0.44
plt count, median (range)	**55**	(5-757)	**21**	(0.6-73)		0.26
plt <20G/L	**4**	29	**9**	36	0.73 (0.30-2.01)	0.73
BM blasts, median (range)	**37**	(8-77)	**29**	(10-92)		0.82
BM blasts <30%	**5**	36	**12**	48	0.72 (0.29-1.76)	0.52
Adverse cytogenetics	**1**	7	**13**	52	0.13 (0.02-0.90)	0.005
monosomal	0	0	**11**	44		0.003
complex	0	0	**10**	40		0.007
genomic alteration						
NPM1	**3**	21	**3**	12	1.5 (0.59-3.82)	0.65
FLT3	**3**	21	**4**	16	1.25 (0.47-3.32)	0.69
ITD	**2**	14	**4**	16		-
TKD	**1**	7	**0**	0		-
IDH (n=37)	**6**	43	**4**	17	2 (0.95-4.21)	0.13
IDH1	**2**	14	**1**	4		-
IDH2	**4**	29	**3**	13		-
TP53 (n=32)	**2**	15	**5**	26	0.69 (0.20-2.42)	0.68
JAK2 (n=28)	**1**	8	**1**	6	1.18 (0.28-5.07)	1
ASXL1 (n=24)	**3**	27	**1**	8	1.87 (0.86-4.09)	0.30
RUNX1 (n=24)	**4**	36	**4**	31	1.14 (0.47-2.78)	1
TET2 (n=24)	**4**	36	**3**	23	1.39 (0.59-3.27)	0.66
DNMT3A (n=24)	**3**	27	**5**	38	0.95 (0.38-2.36)	0.68
SFSR2 (n=24)	**4**	36	**1**	8	2.17 (1.04-4.52)	0.14
RAS/TP53 (n=28)	**2**	18	**7**	54	0.37 (0.10-1.35)	0.10
VEN dose >50% during cycle 1 (n=34)	**11**	79	**19**	83	0.86 (0.32-2.27	1

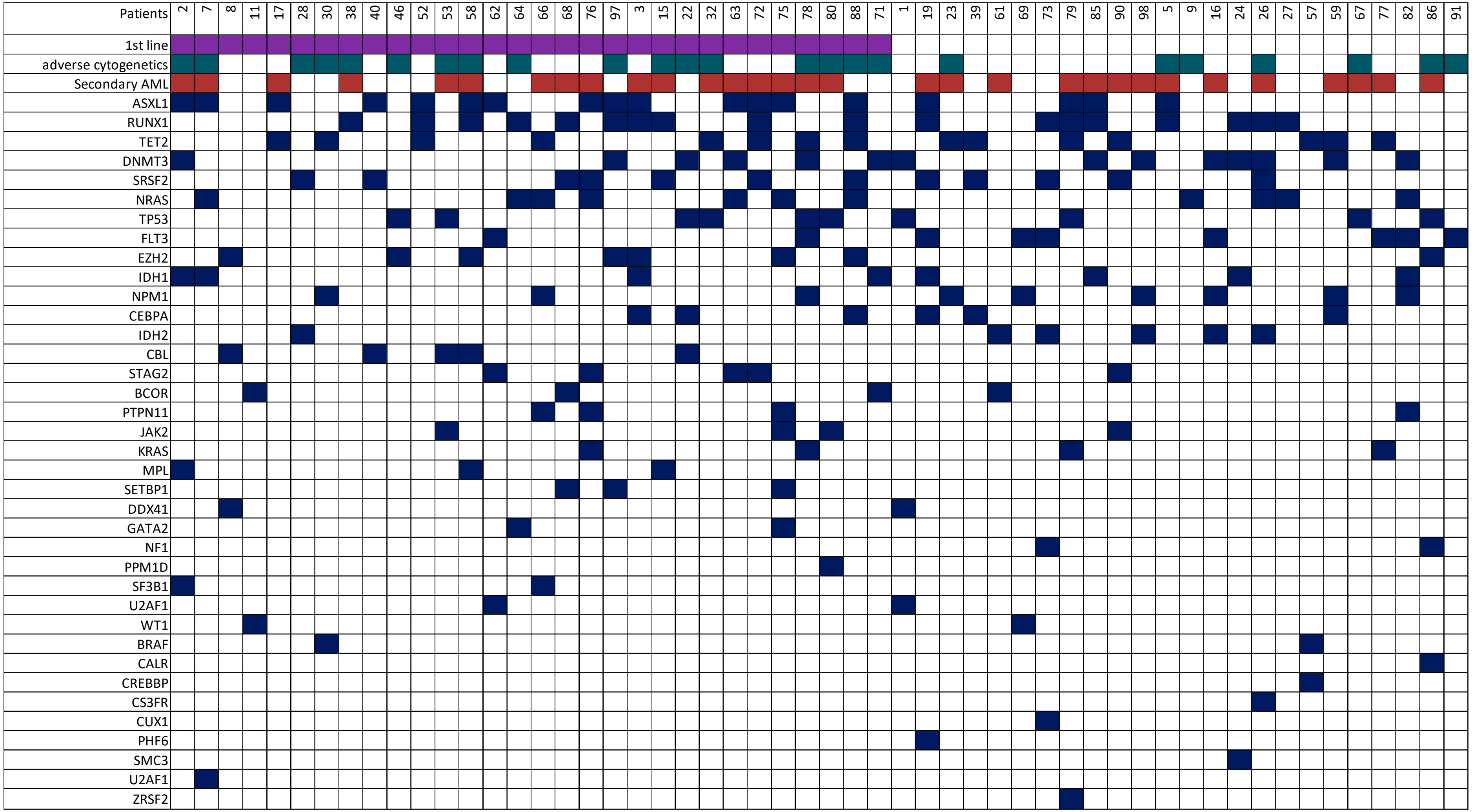

Supplementary Figure S1. Oncoprint showing the set of gene mutations found in the whole VEN-AZA treated population according to treatment groups, cytogenetics and type of AML.

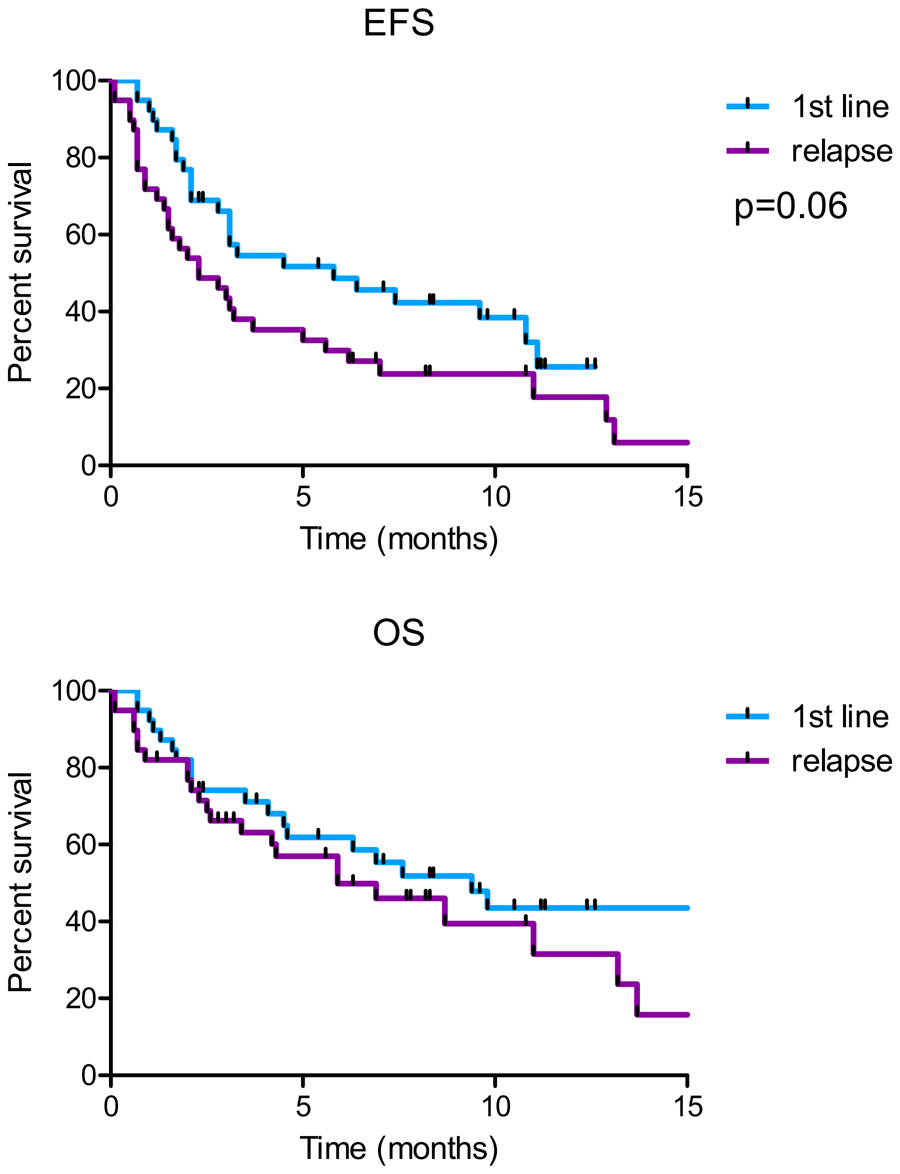

Supplementary Figure S2. Kaplan-Meier analyses showing overall survival and event-free survival in the ND AML group and the R/R group of patients.

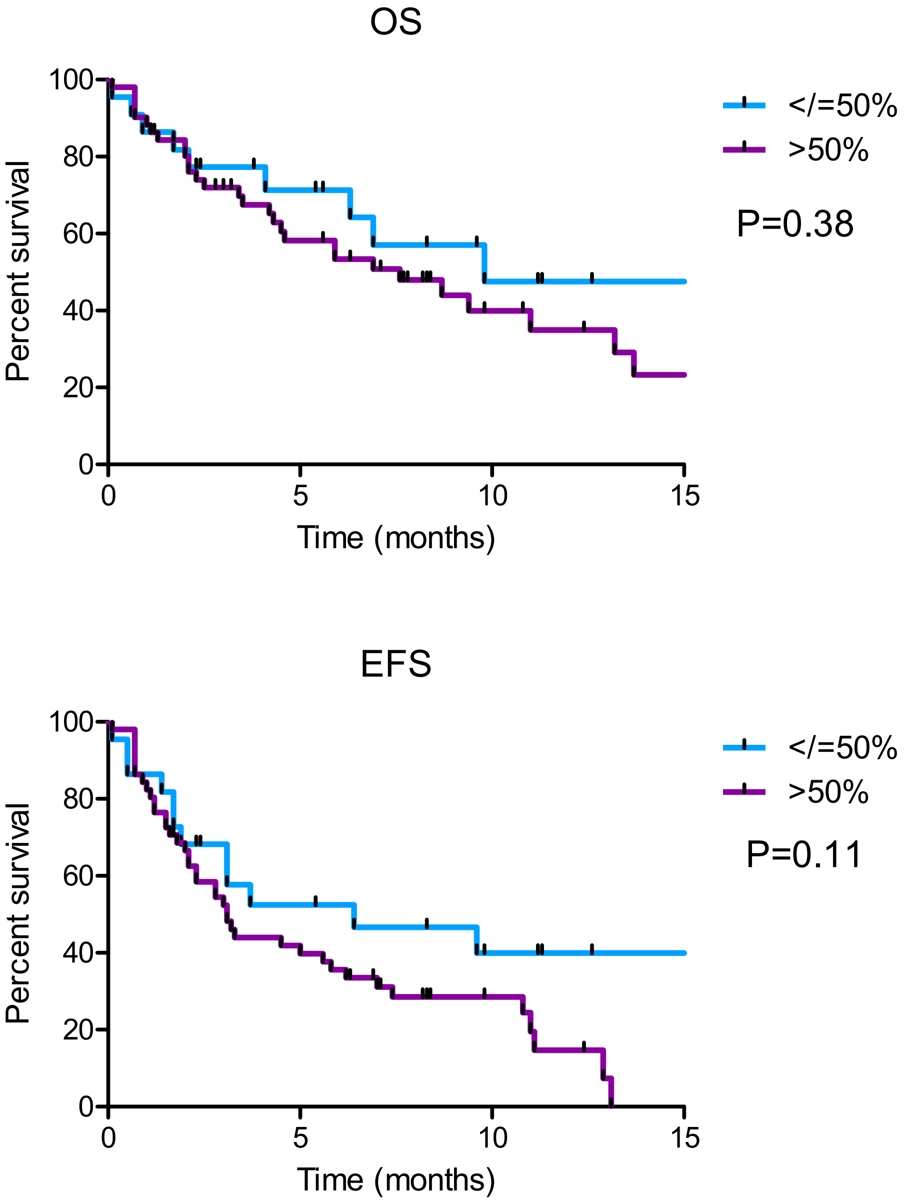

Supplementary Figure S3. Kaplan-Meier analyses showing overall survival (OS) and event-free survival (EFS) according to the percentage of maximum venetoclax dose during the first cycle.
